# Acute intraventricular obstruction: an underrecognized trigger of takotsubo cardiomyopathy in athletes in the setting of endurance activities

**DOI:** 10.21542/gcsp.2026.33

**Published:** 2026-08-31

**Authors:** Kenan Yalta, Muhammet Gurdogan, Yekta Gurlertop

**Affiliations:** Trakya University, Cardiology Department, Edirne, Turkey

Dear Editor,

Takotsubo cardiomyopathy (TTC), also known as takotsubo syndrome, has long been regarded as a stress-induced, transient clinical phenomenon characterized by specific myocardial wall motion abnormalities^1,2^. In their recent article, Katib et al. analysed the characteristics of TTC in endurance athletes presenting in the context of marathon-related events^1^, focusing in particular on the pathogenetic roles of excessive catecholamine release and microvascular dysfunction. We would like to underscore the pathogenetic and clinical implications of acute intraventricular obstruction as an additional mechanism of TTC in this population.

Beyond its well-established association with catecholamine toxicity at the myocardial level, TTC may also arise from intraventricular mechanical factors, including sudden intraventricular obstruction^3,4^. This mechanically triggered form typically develops in response to acute mid-ventricular or outflow tract obstruction in patients with pre-existing hypertrophic conditions such as hypertrophic cardiomyopathy (HCM), hypertensive heart disease, or a ventricular septal bulge^3,4^.

Mechanistically, supply–demand mismatch driven by extreme apical intraventricular pressure produces TTC exclusively in the apical ballooning pattern in such cases^3,4^. Notably, mechanically triggered TTC most often occurs in individuals with borderline or mild myocardial hypertrophy^3,4^. Importantly, the obstruction may be absent at rest yet provoked by manoeuvres that reduce preload and/or afterload and enhance myocardial contractility^3,4^. Provocation tests such as dobutamine stress echocardiography can unmask a latent intraventricular gradient and help guide subsequent management^3,4^.

The ‘athlete’s heart’, by contrast, represents a physiological adaptation characterized by increased ventricular cavity dimensions and mild hypertrophy^5^. On imaging, however, it overlaps substantially with pathological conditions of mild phenotypic expression, including HCM with borderline hypertrophy^5^. Mild hypertensive heart disease or a ventricular septal bulge may likewise masquerade as athlete’s heart, particularly in older athletes^4^. Some TTC cases in this setting may therefore reflect an underlying, undiagnosed pathological myocardial condition; alternatively, such conditions and athlete’s heart may co-exist.

It seems plausible that haemodynamic stressors encountered during endurance activities such as marathon races—dehydration and post-exercise vagotonia (reducing preload and afterload), together with heightened myocardial contractility^4^ driven by extreme catecholamine discharge^1^—could precipitate acute intraventricular obstruction and subsequent TTC in a subset of endurance athletes. Because TTC recurrence may be more likely with this mechanically triggered form^6^, these athletes could be at particular risk of repeat episodes when similar stressors recur during future endurance events.

In this context, we would be interested to learn more about the structural myocardial characteristics of the athletes described—the presence, distribution, and severity of hypertrophy on cardiac imaging—as well as whether any overt intraventricular gradient was documented and whether provocation testing was used to uncover a latent gradient in these cases.

In conclusion, intraventricular mechanical factors, including acute intraventricular obstruction, may act as the actual trigger of TTC in a proportion of endurance athletes, particularly during endurance activities. Timely diagnosis and management of underlying pathological myocardial conditions and associated intraventricular obstruction—whether overt or latent—may help prevent TTC and its recurrence, and potentially improve prognosis in this population.
